# The joint Simon effect depends on perceived agency, but not intentionality, of the alternative action

**DOI:** 10.3389/fnhum.2014.00595

**Published:** 2014-08-05

**Authors:** Anna Stenzel, Thomas Dolk, Lorenza S. Colzato, Roberta Sellaro, Bernhard Hommel, Roman Liepelt

**Affiliations:** ^1^Institute for Psychology, University of MuensterMuenster, Germany; ^2^Department of Psychology, Max-Planck-Institute for Human Cognitive and Brain SciencesLeipzig, Germany; ^3^Research Group: Heterogeneity and Inclusion, Faculty of Human Science, University of PotsdamPotsdam, Germany; ^4^Institute for Psychological Research and Leiden Institute for Brain and Cognition, Leiden UniversityLeiden, Netherlands

**Keywords:** joint Simon effect, joint action, social interaction, stimulus-response compatibility, agency

## Abstract

A co-actor's intentionality has been suggested to be a key modulating factor for joint action effects like the joint Simon effect (JSE). However, in previous studies intentionality has often been confounded with agency defined as perceiving the initiator of an action as being the causal source of the action. The aim of the present study was to disentangle the role of agency and intentionality as modulating factors of the JSE. In Experiment 1, participants performed a joint go/nogo Simon task next to a co-actor who either intentionally controlled a response button with own finger movements (agency+/intentionality+) or who passively placed the hand on a response button that moved up and down on its own as triggered by computer signals (agency−/intentionality−). In Experiment 2, we included a condition in which participants believed that the co-actor intentionally controlled the response button with a Brain-Computer Interface (BCI) while placing the response finger clearly besides the response button, so that the causal relationship between agent and action effect was perceptually disrupted (agency−/intentionality+). As a control condition, the response button was computer controlled while the co-actor placed the response finger besides the response button (agency−/intentionality−). Experiment 1 showed that the JSE is present with an intentional co-actor and causality between co-actor and action effect, but absent with an unintentional co-actor and a lack of causality between co-actor and action effect. Experiment 2 showed that the JSE is absent with an intentional co-actor, but no causality between co-actor and action effect. Our findings indicate an important role of the co-actor's agency for the JSE. They also suggest that the attribution of agency has a strong perceptual basis.

## Introduction

As social beings, we are born into a social environment. Acting in and interacting with our surroundings shapes our behavior and cognition from the early beginning (Prinz, 2012). Previous research on single subjects has enormously improved our understanding of how perception and action are linked (i.e., by sharing common representations), how individuals select task-relevant information, predict upcoming actions, and integrate predicted effects of one's own and others' actions (Wilson and Knoblich, 2005). However, when and to what extent individuals mentally represent their own and others' actions is currently a matter of debate in cognitive science (Liepelt and Prinz, 2011; Guagnano et al., 2013; Welsh et al., 2013a,b).

One of the most popular paradigms to investigate the cognitive processes representing joint action in humans is the joint go/nogo Simon task (Sebanz et al., 2003), in which two individuals share the standard Simon task (Simon and Rudell, 1967; Simon, 1969; see Simon, 1990 for a review). In the standard Simon task, a single participant carries out spatially defined responses, such as left and right key presses, to non-spatial stimulus attributes (e.g., geometric forms) that appear randomly to the left or right side of a central fixation point. Even though stimulus location is completely task-irrelevant, responses are faster when they spatially correspond to the stimulus position, a phenomenon known as the Simon effect (see Hommel, 2011 for an overview). When the same participant responds to only one of the two stimuli by pressing for example the left key, thus rendering the task a go/nogo task, there is typically no Simon effect observable (Hommel, 1996). However, when the same go/nogo task is divided between two co-acting participants, so that each of them performs complementary go/nogo responses next to each other, the Simon effect is re-established across the dyad (Sebanz et al., 2003). This so-called joint Simon effect (JSE) is typically explained by the assumption that interacting individuals automatically co-represent the other person's action (action co-representation), so that performing the Simon task with another person is functionally equivalent to performing the entire standard two-choice Simon task alone (Sebanz et al., 2003, 2005; Knoblich and Sebanz, 2006).

Recent studies, however, showed that a Simon effect can also be observed when replacing the human co-actor in a joint go/nogo Simon task with an event-producing object, like a rotating wheel (Dolk et al., 2011, Experiment 3), a Japanese waving cat or a metronome (Dolk et al., 2013). Based on these findings, a “referential-coding” account has been suggested as an alternative explanation for the JSE. Given that self-generated and other-generated actions are cognitively represented by their sensory consequences, i.e., by using the same kinds of codes (Prinz, 1997; Hommel et al., 2001; Hommel, 2013), the co-actor's action can be considered as just any other event that needs to be differentiated for response coding (Guagnano et al., 2010; Dolk et al., 2011, 2013; Dittrich et al., 2012). As a consequence, the perception of concurrently activated (and thus cognitively represented) social or non-social events that share features with the events that a person produces (i.e., action) introduces a discrimination problem: to enable proper task performance the participant needs to discriminate between the event representations referring to one's own action and all other (concurrently) activated event representations. According to the referential coding account, the action discrimination problem can be resolved by emphasizing processing on event features that discriminate best in a given task context. As the relative spatial location of both alternative actions (distributed to the left and right side) is the most obvious discriminable event feature in the spatial Simon task, it provides a reasonable reference for coding the individual's own action (the single button press) as left or right relative to the alternative event (Dolk et al., 2013). This referential coding of actions in turn can lead to matches or mismatches between the spatial stimulus features and the spatial response features—a necessary condition for Simon effects to emerge (Kornblum et al., 1990; Hommel et al., 2001; Liepelt et al., 2011, 2013; Dittrich et al., 2013; Sellaro et al., 2013). Hence, referential coding assumes that the presence of an alternative action event that shares features with one's own action event is necessary for the JSE to occur, whereas the co-representation of the other's task or task rules is not.

According to the referential coding account, the need to discriminate one's own action event from alternative action events via spatial coding should be stronger the more similar both action events are (Colzato et al., 2012, 2013; Liepelt et al., 2012). In turn, more pronounced spatial coding should lead to a larger JSE (Guagnano et al., 2010; Dolk et al., 2011, 2013). Indeed, there are several recent studies demonstrating that the size of the JSE is modulated by a range of factors that are related to the similarity between the participant and the co-actor. For example, Tsai and Brass (2007) showed that the JSE only emerges when participants share a go/nogo Simon task with a virtual human co-actor (a video of a human hand), but not when the task was shared with a non-human co-actor (a video of a wooden hand). Stenzel et al. (2012) extended these findings by showing that a reliable JSE is observed when a human person shared a task with a real humanoid robot, but only when this person believed that the robot was functioning in a human-like, biologically inspired way, and not when the robot was believed to function like a machine. Both studies suggest that a higher similarity regarding the humanness of the participant and the co-actor leads to a larger JSE. Furthermore, Müller et al. (2011a) showed that actions of in-group members, i.e., a white participant sharing a task with a white virtual co-actor, produced a larger JSE than actions of out-group members, i.e., a white participant sharing a task with a black virtual co-actor. Other components referring to the quality of the interpersonal relationship between both interacting individuals have also been shown to modulate the size of the JSE. In a study by Hommel et al. (2009), for example, the JSE was only present when two actors were in a positive relationship, which might lead participants to perceive the other person as being more similar to themselves. All of these studies provide evidence that a greater similarity between two actors (e.g., regarding their humanness or group membership) leads to a larger JSE.

Atmaca et al. (2011) investigated the role of conceptual similarity between two co-actors defined in terms of similarity regarding intentionality. They used a go/nogo version of the Erikson Flanker task (Eriksen and Eriksen, 1974) that participants performed either alone or together with another person responding to the nogo-stimuli of the participant. In the Flanker task, participants respond to a central target letter that is flanked by task irrelevant letters to its left and right side. The flanking letters are either the same as the target letter (e.g., SSSSS, compatible trial) or different (e.g., HHSHH, incompatible trial). Participants showed a larger Flanker effect (i.e., faster responses in compatible than incompatible trials) when performing the go/nogo Flanker task together with another person than when performing the same task alone—a phenomenon known as the joint Flanker effect. When performing the task with a co-actor whose response button was controlled by a computer (unintentional co-actor condition) the Flanker effect was smaller than when performing the task with a co-actor who actively controlled her response button (intentional co-actor condition) (Atmaca et al., 2011, Experiment 4). Atmaca and colleagues suggested that humans only form shared task representations when perceiving another person as acting intentionally. However, recently Dolk et al. (2014) could show that—just like for the JSE—a joint Flanker effect can be induced even if the human co-actor is replaced by an event-producing object (a Japanese waving cat). The logic applied to explain the joint Flanker effect with referential coding goes as follows. As actions are coded on more dimensions in the presence of an event-producing human or object than in its absence, response competition is increased, and hence behavioral effects that rely on response competition (like the Flanker effect) are enhanced.

In line with the findings by Atmaca et al. (2011) other studies have suggested that the intentionality of a co-actor is a key conceptual feature modulating joint action effects with larger effect sizes for intentional than unintentional co-actors (Tsai et al., 2008; Müller et al., 2011b; Stenzel et al., 2012). The concept of intentionality comprises components like belief, desire, intention and awareness (Malle and Knobe, 1997). All of these mental states can be ascribed to other biological agents or technical systems that function according to biologically inspired algorithms, but clearly not to objects. The assumption that joint action effects can only be found for intentional co-actors is at odds with the outlined findings showing emerging Simon or Flanker effects for non-human event-producing objects (Dolk et al., 2011, Experiment 3; Dolk et al., 2013, 2014), and raises doubts in the crucial role of intentionality for joint action. Whereas intentionality, by definition, cannot be ascribed to objects, objects can be identified as the physical cause of an (action) effect (e.g., a ticking metronome is the causal source of peep tones or a Japanese cat the initiator of an arm movement). The process of identifying an agent as the initiator or causal source of an action has been defined as agency (Gallagher, 2000). In the present manuscript, we define the process of perceiving the physical causality between an initiator of an (action) effect and the effect, independently of whether the initiator is a human agent, non-human agent or object as the minimum-criterion of agency. The work of Albert Michotte (1963) suggests that ascribing causality to two events depends on perceptual features. In his famous launching effect, an object (the so called launcher) moves in the direction of another object, stops when making contact with it, whereupon the other object starts to move. Whether the first object is regarded as causing the movement of the second object has been found to depend on different perceptual parameters like the speed with which both objects move, the direction they move, and the time interval between the movement offset of the first object and the movement onset of the second. In light of these findings, and the finding that identifying an agent as the cause of an action effect is particularly likely when action and action effect appear in close temporal proximity (Haggard et al., 2002; Moore and Haggard, 2008), the ascription of agency could critically rely on perceiving the causality between initiator and action effect. For example, identifying a person as the initiator of a button press could rely on seeing how the finger of the person moves down in order to press the button.

In many previous studies that investigated the effects of intentionality on joint action, intentionality and agency were confounded. That is, intentional co-actor's could be clearly perceived as being the initiator of the action effect, while unintentional co-actor's were clearly not the initiator of the action effect. Related to this problem, intentional and unintentional experimental conditions did not only differ in conceptual features (i.e., intentionality), but often also regarding perceptual features. For example, in the study by Atmaca et al. (2011) the response button of the intentional co-actor differed in size, shape, and probably also in sound from the response button of the unintentional co-actor. In both conditions, response buttons were permanently visible to participants while performing the task. An open question is whether intentionality alone, in the absence of agency, can modulate joint action effects like the JSE.

In the present study, we aimed to disentangle the role of intentionality and agency in modulating the JSE. Further, we controlled for differences in perceptual features between manipulations of intentionality. In Experiment 1, we aimed to replicate the findings of Atmaca et al. (2011) for the joint go/nogo Simon effect while controlling for perceptual differences between conditions during task performance. We compared JSEs between a condition in which the co-actor intentionally controlled a response button and could be perceived to be the agent of the action (agency+/intentionality+ condition) and a condition in which the co-actor acted unintentionally and was clearly not the agent of the action, because the co-actor's hand rested on a response button that passively moved up and down on its own as triggered by the computer, so that the physical causality between the co-actor and the button movement was clearly disrupted (agency−/intentionality− condition). During the experiment, we controlled for perceptual differences (visual and auditory action effects) between both conditions by using boxes covering the hands of both persons and letting persons wear earplugs. In Experiment 2, we again included a control condition in which the co-actor acted unintentionally and was not the agent of the computer controlled button press (agency−/intentionality− condition). Performance in this condition was compared to a condition in which the co-actor was believed to control the response button with a Brain-Computer Interface (BCI) instead of manual responses, so that the co-actor could be regarded as intentionally controlling the response button, but the causal relationship between co-actor and action effect could not be perceived (agency−/intentionality+).

For Experiment 1, agency and intentionality make similar predictions. This would be a conceptual replication of the findings of Atmaca et al. (2011) for the JSE (JSE_agency+/intentionality+_ > JSE_agency−/intentionality−_). For Experiment 2, the predictions do crucially differ for intentionality and agency. If the co-actor's intentionality is the underlying source for modulating joint action effects, we predicted a larger JSE when the co-actor acts intentionally than when the co-actor does not act intentionally (JSE_agency−/intentionality+_ > JSE_agency−/intentionality−_). If, however, agency is the modulating factor of the JSE, we expect no differences in JSEs between conditions (JSE_agency−/intentionality+_ = JSE_agency−/intentionality−_).

## Experiment 1

The aim of Experiment 1 was to investigate whether a co-actor's agency and intentionality modulate the size of the JSE. Different from Atmaca et al. (2011), we controlled for perceptual differences between experimental conditions while participants were performing the task. Participants performed a joint go/nogo Simon task with a co-actor who either actively controlled a response button and could clearly be perceived as the agent of the button press (agency+/intentionality+ condition) or whose response button was controlled by the computer so that the co-actor was not the agent of the button press (agency−/intentionality− condition). We expected a larger JSE in the agency+/intentionality+ condition than in the agency−/intentionality− condition.

### Methods

#### Participants

A total of 32 healthy volunteers participated in Experiment 1. Sixteen participants were randomly assigned to the agency+/intentionality+ condition (15 female, mean age = 23.3 years, *SD* = 4.8 years) and 16 participants to the agency−/intentionality− condition (12 female, mean age = 21.7 years, *SD* = 2.8 years). All participants were right-handed, had normal or corrected-to-normal vision, were naive with regard to the hypothesis of the experiment, and received compensation for their participation.

#### Stimuli and apparatus

As stimuli we used a white square and a white diamond (2.2 × 2.2°, horizontal × vertical visual angle) on a black background, which were presented 5.4° to the left or right of a centrally presented white fixation cross (0.9 × 0.9°). All stimuli were displayed on an 18-inch CRT monitor at a viewing distance of approximately 60 cm.

For the agency+/intentionality+ condition we used two conventional response keys (one for the participant and the other for the co-actor). For the agency−/intentionality− condition we used one conventional response key for the participant and one response key that could be moved up and down by a trigger signal from the computer for the co-actor. Both response keys were placed 5 cm in front of the monitor and 27 cm from the midline of the monitor.

#### Task and procedure

**Figure 1:**
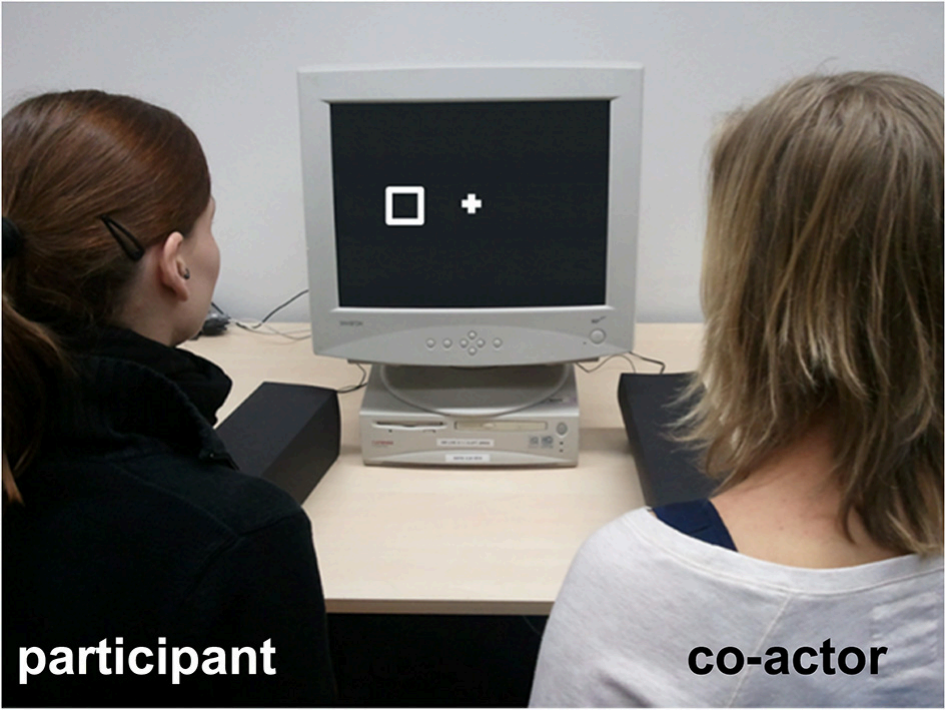
**Experimental setup used in Experiment 1**. The participant (sitting on the left side of the monitor) shared a joint go/nogo Simon task with a co-actor (confederate) who either intentionally controlled a response button, and could be perceived as the initiator of the button press (agency+/intentionality+ condition) or whose response button was controlled by the computer so that the co-actor was not the initiator of the button press and did not respond intentionally (agency−/intentionality− condition). Perceptual differences between the response button of the actively responding co-actor and the computer controlled response button were controlled for during task performance by covering response hands and letting both persons wear earplugs, so that the setup shown on the picture applies to both, the agency+/intentionality+ and the agency−/intentionality− condition.

The participant was always seated on the left side of the monitor and the co-actor (a confederate) on the right side (Figure 1). Both persons were asked to place their right index finger on the response button in front of them. The participant responded to the square, whereas the co-actor responded to the diamond. Participants either performed the task with a co-actor who actively controlled the response button (agency+/intentionality+ condition) or with a co-actor whose response button was controlled by the computer via trigger signals (agency−/intentionality− condition) (Figure 1). In the latter condition, the co-actor passively placed her index finger on the response button, which was automatically pulled down every time it was the co-actor's turn to respond. The response latency of the computer controlled response button varied randomly between 280, 320, and 360 ms. The participant actively controlled his/her response button in both conditions. Participants were randomly assigned to conditions.

As the conventional and the computer controlled response button differed in size and shape, response keys were covered with black boxes before the experimental task started to control for visual differences between both conditions during task performance (Figure 1). In addition, the participant and the co-actor wore earplugs in both conditions in order to control for the different sounds of the conventional and the computer controlled response key.

The instruction given to the co-actor was audible to participants in both conditions. In the agency+/intentionality+ condition, the instruction for the co-actor was to press the response button whenever a diamond appeared on the screen. In the agency−/intentionality− condition, the co-actor was instructed to position her response finger on the response button located in front of her. The co-actor was informed that the stimulus computer sent a trigger signal to start the movement of the button whenever a diamond appeared thereby controlling the response button. To familiarize participants with the task, the experiment started with a short instruction phase including the presentation of the two stimuli, their assignment to both actors, as well as the presentation of the conventional and the computer controlled response key. For the practice phase, the box that covered the hands during the experiment was removed so that the participant could clearly see that the co-actor actively responded in the agency+/intentionality+ condition, whereas the response button moved on its own when receiving a signal from the computer in the agency−/intentionality− condition.

There were two blocks of 64 trials separated by short breaks of 2 min. The two target stimuli appeared equally often in the left and right location which resulted in a total of 32 Stimulus-Response (S-R) compatible trials and 32 S-R incompatible trials for each person. The order of trials was randomized. Each trial began with the presentation of the fixation cross for 1000 ms. Afterwards the target stimulus was displayed together with the fixation cross for 150 ms. The response had to be given within a time interval of 1800 ms following stimulus offset during which the fixation cross was displayed. Following a response, feedback about the accuracy was provided for 300 ms: correct responses were followed by the fixation cross, incorrect responses by the word “Fehler” (error), and too slow responses by “zu langsam” (too slow). In the inter-trial-interval the fixation cross was displayed for 1000 ms.

As a manipulation check verifying that there is a difference between both conditions regarding the intentionality attributed to the co-actor, participants rated the items “The other person acted intentionally” (Item 1) and “The other person decided actively when to respond to a stimulus” (Item 2) after the experiment. Both items were rated on a five-point Likert scale ranging from 0 (= I strongly disagree) to 4 (= I strongly agree) with 2 indicating neither agreement nor disagreement. Participants in the agency+/intentionality+ condition showed significantly higher mean rating scores for both items than participants in the agency−/intentionality− condition [Item 1: 2.4 vs. 0.8, *t*_(30)_ = 3.64, *p* = 0.001; Item 2: 3.1 vs. 0.7, *t*_(30)_ = 7.24, *p* < 0.0001].

### Results

In accordance with previous studies (Röder et al., 2007; Liepelt et al., 2011), we excluded all trials in which responses were incorrect (1.5%), faster than 150 ms or slower than 1000 ms (0%) prior to the statistical analysis of reaction times (RTs). Responses were coded as compatible (stimulus ipsilateral to the correct response side) or incompatible (stimulus contralateral to the correct response side). We calculated a repeated measures analysis of variance (ANOVA) for RTs and errors with the within-subjects factor compatibility (compatible, incompatible) and the between-subjects factor condition (agency+/intentionality+, agency−/intentionality−). The JSE was calculated by subtracting mean RTs in compatible trials from mean RTs in incompatible trials. Additionally, we calculated Bayesian probabilities associated with the occurrence of the null (H_0_) and alternative (H_1_) hypotheses, given the observed data (see Wagenmakers, 2007; Masson, 2011). This method allows making inferences about both significant and non-significant effects by providing the exact probability of their occurrence, with values ranging from 0 (i.e., no evidence) to 1 (i.e., very strong evidence; see Raftery, 1995 for a coarse classification).

#### Reaction times

**Figure 2:**
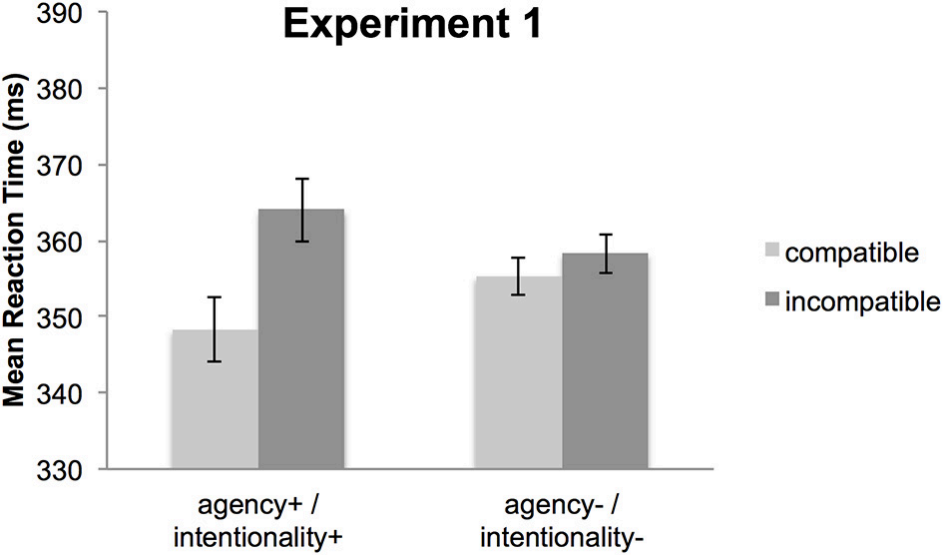
**Mean reaction times for Experiment 1**. Depicted are compatible (light gray) and incompatible (dark gray) trials for the agency+/intentionality+ condition (left panel) and the agency−/intentionality− condition (right panel). Error bars represent standard errors of the mean differences (Pfister and Janczyk, 2013).

The 2 × 2 ANOVA revealed a significant main effect of compatibility, *F*_(1, 30)_ = 14.22, *p* = 0.001, partial η^2^ = 0.32, *p*(H_1_|D) > 0.99, indicating faster responses for S-R compatible trials (352 ms) than incompatible trials (361 ms). More importantly, the compatibility effect differed for the two conditions as indicated by a significant interaction of compatibility and condition, *F*_(1, 30)_ = 6.55, *p* = 0.02, partial η^2^ = 0.18, *p*(H_1_|D) = 0.99. Newman-Keuls *post-hoc* analyses revealed that the difference between compatible and incompatible trials was significant in the agency+/intentionality+ condition (16 ms, *p* < 0.001, *d* = 0.93) but not in the agency-/intentionality- condition (3 ms, *p* = 0.40, *d* = 0.29) (Figure 2). There was no significant main effect of condition, *F*_(1,30)_ < 1, *p* = 0.96, partial η^2^ < 0.001, *p*(H_0_|D) = 0.85.

#### Error rates

The main effects of compatibility, *F*_(1, 30)_ < 1, *p* = 0.90, partial η^2^ = 0.001, *p*(H_0_|D) = 0.85, and condition, *F*_(1, 30)_ < 1, *p* = 0.68, partial η^2^ = 0.006, *p*(H_0_|D) = 0.84, as well as the interaction of compatibility and condition, *F*_(1, 30)_ = 1.40, *p* = 0.25, partial η^2^ = 0.04, *p*(H_0_|D) = 0.73, were not significant.

### Discussion

In Experiment 1, we observed a significant JSE when interacting with a co-actor who actively controlled the response button (agency+/intentionality+ condition), but found no significant JSE when the co-actor's response button was controlled by the computer (agency−/intentionality− condition). The JSE in the agency+/intentionality+ condition was significantly enlarged as compared to the agency−/intentionality− condition, conceptually replicating the findings of Atmaca et al. (2011) for the joint go/nogo Simon effect. So, even in the absence of perceptual differences between co-acting agents, past perception of physical causality between co-actor and action effect and/or the ascription of intentionality to the co-actor appear to be sufficient in modulating the size of the JSE. Given that intentionality and agency were clearly confounded in this experiment, in a second experiment we aimed at separating both aspects by varying the co-actor's intentionally between conditions while keeping the physical causality between co-actor and action effect constant.

## Experiment 2

**Figure 3:**
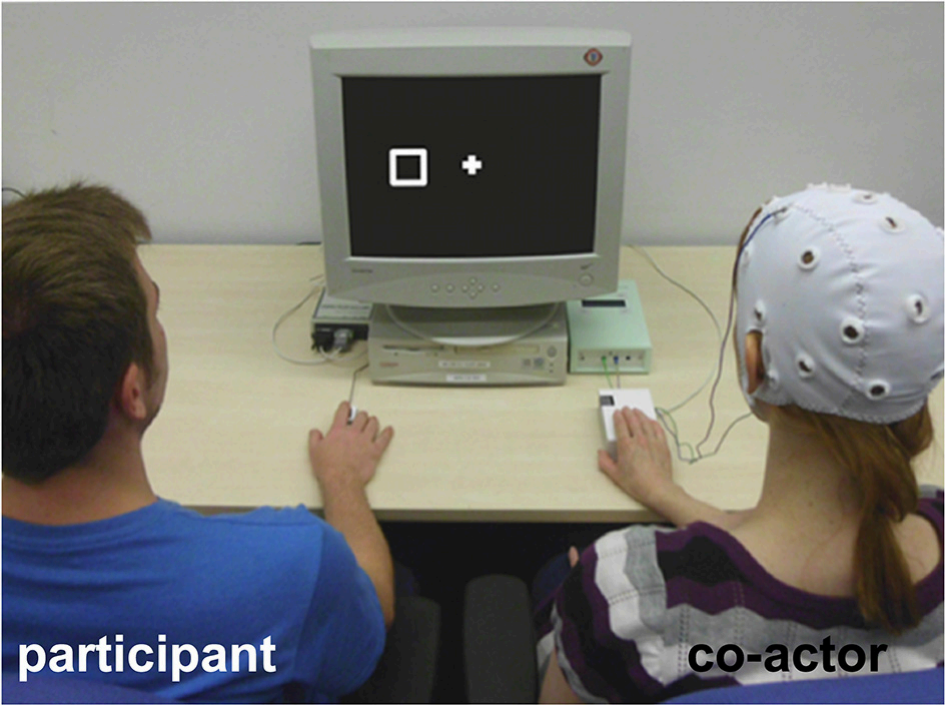
**Experimental setup used in Experiment 2**. The participant (left side) shared a joint go/nogo Simon task with a co-actor (confederate, right side) wearing an EEG cap with electrodes attached to the motor cortex, and placing the finger underneath the moving part of a response device. The participant was either told that the confederate intentionally controlled the response button via a BCI so that the causal relationship between co-actor and action effect was not perceivable (agency−/intentionality+ condition) or that the response button was controlled by the computer (agency−/intentionality− condition). As the same response button was used for the co-actor in the agency−/intentionality+ and the agency−/intentionality− condition, the setup shown on the picture applies to both conditions.

The aim of Experiment 2 was to disentangle the effects of intentionality and agency on the JSE. We included a condition, in which the co-actor was believed to intentionally control the response button, but the causal relationship between agent and action effect could not be perceived, so that physical causality was disrupted (agency−/intentionality+ condition). In this condition, the co-actor was equipped with a cap used to measure electroencephalography (EEG) activity including two electrodes over the motor cortex. Participants were made to believe that the co-actor controlled the response button via a BCI by generating motor potentials whenever it was the co-actor's turn to respond. We again compared the size of the JSE in this condition to a condition in which the co-actor passively placed her finger on a computer controlled response button (agency−/intentionality− condition). In this condition, participants were told that the co-actor was wearing an EEG cap in order to measure electrical potentials in a motor observation task. To fully eliminate any perceptual differences between the agency−/intentionality+ and the agency−/intentionality− condition, the co-actor's response button was identical for both conditions (see Figure 3, co-actor side), so that only the belief about the co-actor's intentionality differed between conditions.

If the co-actor's intentionality modulated the size of the JSE in the previous experiment, we would expect a similar response time pattern as in Experiment 1 with a larger JSE when the co-actor acts intentionally than when acting unintentionally (JSE_agency−/intentionality+_ > JSE_agency−/intentionality−_). If, however, the modulation of the JSE in Experiment 1 was driven by agency, we expect JSEs of comparable size in both conditions (JSE_agency−/intentionality+_ = JSE_agency−/intentionality−_).

### Method

#### Participants

Thirty-two new healthy volunteers participated in Experiment 2. Sixteen participants were assigned to the agency−/intentionality+ condition (13 female, mean age = 23.5 years, *SD* = 3.7 years) and 16 to the agency−/intentionality− condition (12 female, mean age = 23.9 years, *SD* = 2.3 years). All participants fulfilled the same criteria as participants in Experiment 1 and were treated in the same way.

#### Stimuli and apparatus

Stimuli and apparatus were the same as in Experiment 1. The co-actor (a confederate) wore an EEG cap equipped with one electrode over the left and one over the right motor cortex (Figure 3). The cable of the electrodes was connected to a box placed on the right side of the monitor (Figure 3). Participants were told that this box was connected to the stimulus computer, analyzing the electrical signals measured over the motor cortex.

#### Task and procedure

Task and procedure were the same as in Experiment 1 with the following exceptions concerning the co-actor. In both, the agency−/intentionality+ and agency−/intentionality− condition, the computer controlled response button was placed in front of the co-actor, and the co-actor passively placed her right hand on the response device (Figure 3). Note that in both conditions the finger of the co-actor was positioned about 2 cm below the moving part of the response device (Figure 3) in order to avoid any movements of the co-actor's finger which could have led to the false assumption that the co-actor was controlling the response button by finger movements. As the agency−/intentionality+ and the agency−/intentionality− condition were therefore perceptually identical, the left and the right response button were visible during the entire experiment. To manipulate the agency of the co-actor, we used a belief manipulation: in the agency−/intentionality+ condition, participants were told that the co-actor controlled the response button via a BCI. They were told that the co-actor had undergone multiple training sessions to be able to generate motor potentials by imagining a button press with the right index finger. Whenever these motor potentials measured over the motor cortex exceeded a certain threshold, a signal was sent to the response device as a starting signal to move. Hence, participants were led to believe that the co-actor intentionally controlled the response device via brain signals, but the physical causality between co-actor and action effect could not be perceived as the co-actor's finger was clearly placed below the response button on the response device. In the agency−/intentionality− condition, participants were told that the computer controlled the response button. As a cover story to explain why the co-actor wore an EEG cap during in the agency−/intentionality− condition, we explained that the study was about action observation of human and non-human actions, and that the goal of the study was to compare motor potentials elicited by the observation of a human response (the button press of the participant) to those elicited by the observation of a non-human response (the computer controlled response device). In both conditions, the experimenter presented pictures while instructing in order to support the belief manipulation. In the agency−/intentionality+ condition, participants were shown a schematic illustration of the BCI principle and a picture of a child using a BCI to operate a cursor on a monitor. In the agency−/intentionality− condition, the participant and the co-actor were shown a schematic illustration of an electrode over the cortex to explain the principle of measuring evoked potentials. In addition, a picture was shown in which a man equipped with an EEG cap observed a picture of a hand posture. Actually, in both conditions the response button was controlled by the computer.

We used the same manipulation check as in Experiment 1. Participants in the agency−/intentionality+ condition showed significantly higher rating scores for both items than participants in the agency−/intentionality− condition [Item 1: 2.3 vs. 0.8, *t*_(30)_ = 3.22, *p* = 0.003; Item 2: 2.6 vs. 0.8, *t*_(30)_ = 3.75, *p* = 0.001] indicating that the belief manipulation was successful.

### Results

For the statistical analyses of RTs, we again excluded all trials in which responses were incorrect (0.9%), faster than 150 ms or slower than 1000 ms (0%). We calculated a repeated measure ANOVA for RTs and errors with the within-subjects factor compatibility (compatible, incompatible) and the between-subjects factor condition (agency−/intentionality+, agency−/intentionality−). As in Experiment 1, Bayesian probabilities associated with the occurrence of H_0_ and H_1_ were calculated.

#### Reaction times

**Figure 4:**
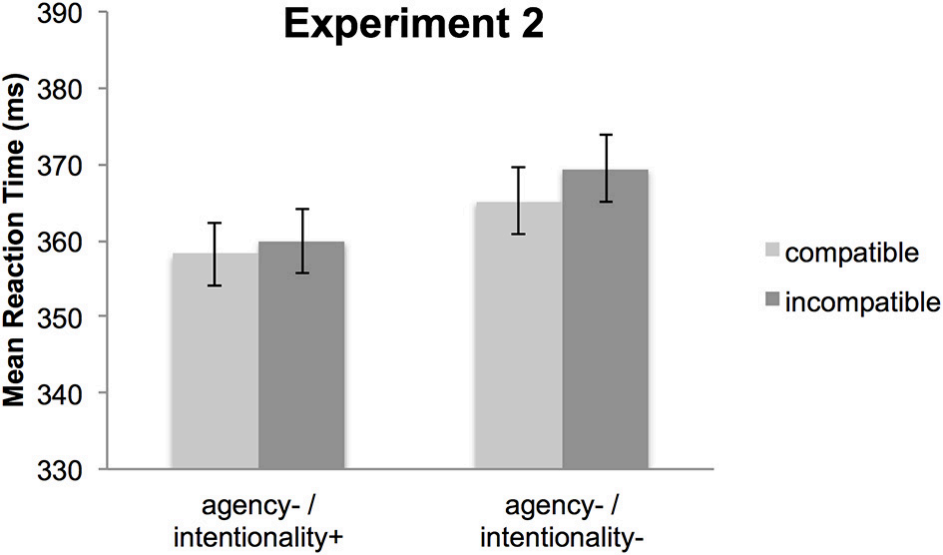
**Mean reaction times for Experiment 2**. Depicted are compatible (light gray) and incompatible (dark gray) trials for the agency−/intentionality+ condition (left panel) and the agency−/intentionality− condition (right panel). Error bars represent standard errors of the mean differences (Pfister and Janczyk, 2013).

The main effect of compatibility was not significant, *F*_(1, 30)_ < 1, *p* = 0.33, partial η^2^ = 0.03, *p*(H_0_|D) = 0.77, indicating comparable response times for S-R compatible trials (362 ms) and incompatible trials (365 ms) (Figure 4). The main effect of condition, *F*_(1, 30)_ < 1, *p* = 0.54, partial η^2^ = 0.01, *p*(H_0_|D) = 0.82, as well as the interaction between compatibility and condition, *F*_(1, 30)_ < 1, *p* = 0.69, partial η^2^ = 0.006, *p*(H_0_|D) = 0.84, were not significant.

#### Error rates

The main effect of compatibility, *F*_(1, 30)_ = 4.2, *p* = 0.049, partial η^2^ = 0.12, but *p*(H_1_|D) = 0.60, was significant indicating fewer errors for compatible (0.6%) than incompatible trials (1.3%). The main effect of condition, *F*_(1, 30)_ < 1, *p* = 0.78, partial η^2^ = 0.003, *p*(H_0_|D) = 0.84, as well as the interaction of compatibility and condition, *F*_(1, 30)_ < 1, *p* = 0.77, partial η^2^ = 0.003, *p*(H_0_|D) = 0.84, were not significant.

### Discussion

In line with the results from Experiment 1, no JSE was observed when participants assumed that the co-actor's response button was controlled by the computer (agency−/intentionality− condition). Importantly and different from Experiment 1, no JSE was induced by the co-actor who intentionally controlled her response button via a BCI, but the causal relationship between agent and action effects could not be perceived. As the results of the post-experimental ratings suggest that our belief manipulation was successful (i.e., participants stated that the co-actor acted intentionally in the agency−/intentionality+ condition), we conclude that agency—perceiving the co-actor as being the causal source of an action effect—seems to be a critical factor for the JSE.

## General discussion

In the present study, we aimed to disentangle the role of agency and intentionality for the JSE. Whereas differences in intentionality were confounded with differences in agency in previous studies (e.g., Tsai and Brass, 2007; Atmaca et al., 2011), we aimed to solely test the effects of intentionality on joint action.

In Experiment 1, actively responding participants performed a joint go/nogo Simon task with a co-actor who responded actively or a co-actor whose response button was controlled by the computer. While controlling for perceptual differences between both conditions during task performance, participants had the opportunity to clearly perceive how the response button was controlled in each condition prior to task performance. When the participant and the co-actor both acted intentionally and physical causality could be perceived the JSE was highly significant, whereas no JSE was observed when the co-actor did not respond intentionally and physical causality was disrupted. In Experiment 2, we again included a condition in which the co-actor's response button was controlled by the computer, and the co-actor clearly was not the agent of the action effect. Performance in this condition was compared to a condition in which the co-actor controlled the response button intentionally via a BCI placing the response finger clearly below the button, so that the physical causality between co-actor and action effect was disrupted. In line with Experiment 1, we observed no JSE when the co-actor was believed to respond unintentionally and was not the agent of the button press (agency−/intentionality− condition). However, different from Experiment 1 no JSE was found for the intentional co-actor when participants did not perceive that the co-actor caused the button press, even though post-experimental questionnaires indicated that the co-actor was believed to intentionally control the response button via the BCI. These results suggest that perceiving the co-actor at least once as being the causal source of responses seems to be a necessary prerequisite for the emergence of a JSE in a real (vis-à-vis) interaction, and point to the co-actor's agency as a modulating factor for joint action effects. Intentionality alone does not seem to be sufficient to induce a JSE. Only when physical causality between co-actor and action effect can be perceived, a JSE seems to be observed.

In the present study, we did not include a condition in which the co-actor was the causal source of action effects, but acted unintentionally (i.e., a agency+/intentionality− condition). However, this condition has already been tested in previous studies using different event-producing objects that replaced the co-actor in joint action tasks (e.g., Dolk et al., 2011, 2013, 2014). By definition, intentionality cannot be ascribed to objects, but the objects used in these studies were the causal source of the respective (action) effects (e.g., the tone originated from the metronome), so physical causality between object and action effect was clearly present. Under these conditions a JSE has consistently been found, suggesting that agency can induce a JSE in the absence of intentionality.

Our findings are in line with the referential coding account (Dolk et al., 2011, 2013) predicting that a higher similarity (conceptual and perceptual) between alternative action events should lead to a larger JSE. When the participant's and the co-actor's action events were highly similar with regards to their conceptual features (i.e., both actors responded intentionally) and perceptual features (i.e., physical causality could be perceived for both actors) the JSE was highly significant, whereas no JSE was observed when both persons were dissimilar (i.e., the participant still acted intentionally and physically caused the action effect, but the co-actor acted unintentionally and physical causality was disrupted). A higher similarity between the action events of both actors induced a (stronger) discrimination problem, which could be resolved by emphasizing spatial action features leading to a JSE. In contrast, the need to emphasize discriminable action features seemed to be weaker and cognitively less demanding when the action features of the co-actor were clearly distinct. Interestingly, conceptual features alone (i.e., intentionality) did not induce a JSE, whereas similarity regarding perceptual features (i.e., visible physical causality) seems to be sufficient to induce a JSE (Dolk et al., 2011, 2013).

A study by Stenzel et al. (2012) compared the size of the JSE in a joint Simon task shared with a robot that was believed to function in a human-like way to a task shared with a robot believed to be controlled by the stimulus computer, and thus to function like a machine. In line with the present results, a JSE was present for the human-like robot which was believed to respond intentionally (i.e., the decision to respond was calculated by a neural network integrated in the robot's body on the basis of visual information recorded by the robot's cameras), and for which agency was clearly present (i.e., participants could see how the robot moved its finger down to press the response button). In contrast, for the machine-like robot, to which intentionality could not be ascribed (i.e., participants believed that responses were controlled by a fixed sequence of trigger signals originating from the stimulus computer, which was located outside of the robotic agent), no JSE was observed. As the machine-like robot condition was perceptually identical to the human-like robot condition, participants could see how the finger of the machine-like robot moved down to press the button, so that—based solely on these visual information—agency could also be attributed to the machine-like robot, and hence a JSE should have been observed. However, due to the belief manipulation used in the robot study the causal source of the action was spatially shifted away from the machine-like robot to the stimulus computer, which was located outside of the robot, so that based on this knowledge the robot could not be regarded as the causal source of the action. That is, the verbal instruction given to the participant disrupted the physical causality between robot and action effect, which could explain why no JSE was observed for this robot. From the perspective of the present findings, the finding of a JSE for the human-like robot may be better interpreted as the result of an interplay between intentionality and agency, and not solely on intentionality.

An interesting question for future research is whether gaining experience with a BCI would lead to a JSE in an agency−/intentionality+ condition like the one used in Experiment 2. As most people are currently not experienced in using BCIs, it might be rather difficult to get a notion of how a person using a BCI accomplishes it to control a response device solely based on the information provided by the experimenter. This might be especially hard because of two reasons. First, the ascription of physical causality seems to be strongly perceptually grounded (Michotte, 1963), so that the ascription of agency might be hard when the causality between agent and action effect cannot be perceived. Second, for human actors with whom we usually interact on a daily basis from very early age on, and on the basis of being humans ourselves, we had the opportunity to develop a fixed notion about how humans usually control actions, so that it might be difficult to get rid of this notion and develop a new understanding of action control using newly developed methods such as BCI. If participants would gain more experience in using BCIs themselves, i.e., gaining perceptual experience in controlling a given device with brain signals, knowing about the physical causality of such action-effect relations may foster recognizing a person using a BCI as being the initiator of action effects even in the absence of any directly perceived causality. This in turn might induce a JSE in a BCI condition like the one used in Experiment 2.

Based on the present findings we would argue that the concept of agency might be better suited than the concept of intentionality to explain the modulatory findings of the JSE in the previous study and potentially in previous studies using human co-actors (e.g., Sebanz et al., 2003, 2005; Vlainic et al., 2010; Liepelt et al., 2011), non-human co-actors (Müller et al., 2011b; Stenzel et al., 2012), and objects (Dolk et al., 2011, Experiment 3; Dolk et al., 2013, 2014). In contrast to intentionality, the attribution of agency—identifying the causal source of an (action) effect—can be applied to biological agents as well as to non-biological agents and objects (Pickering, 1995). As long as a human, a robot or an object is the causal source of an (action) effect and this causal relationship is clearly perceivable, and not otherwise disrupted by instruction, a joint action effect can be observed. Of course this is not to say that agency is the only modulating factor of joint action effects. Other factors that surely determine the size of the JSE refer to the degree of similarity on a perceptual level between the participant's and the co-actor's action effects (Sellaro et al., under revision) or the degree of similarity on more abstract levels like the personal relationship between both actors (Hommel et al., 2009).

Taken together, the results of the present study suggest that a co-actor's agency has a reliable influence on the joint go/nogo Simon effect. Further, our results suggest that in order to ascribe agency to an initiator of action effects, the causal relationship between the initiator and the action effect must be perceived (Michotte, 1963) suggesting a perceptual grounding of physical causality ascription.

## Author contributions

Roman Liepelt, Anna Stenzel, and Bernhard Hommel contributed to the conception and design of the work. Anna Stenzel and Roberta Sellaro analyzed the data. Roman Liepelt, Anna Stenzel, Thomas Dolk, Lorenza S. Colzato, Roberta Sellaro, and Bernhard Hommel contributed to the interpretation of the work. Anna Stenzel and Roman Liepelt wrote the initial draft of the manuscript, which was critically revised by Roberta Sellaro, Thomas Dolk, Lorenza S. Colzato, and Bernhard Hommel. All authors approved the final version of the manuscript and are fully accountable for all aspects of the work.

### Conflict of interest statement

The authors declare that the research was conducted in the absence of any commercial or financial relationships that could be construed as a potential conflict of interest.
